# Practices to Conserve Pollinators and Natural Enemies in Agro-Ecosystems

**DOI:** 10.3390/insects12010031

**Published:** 2021-01-05

**Authors:** Filitsa Karamaouna, Josep A. Jaques, Vaya Kati

**Affiliations:** 1 Scientific Directorate of Pesticides Assessment and Phytopharmacy, Benaki Phytopathological Institute, 7 Ekalis str., 145 61 Kifissia, Greece; 2 Departament de Ciències Agràries i del Medi Natural, Universitat Jaume I, Campus del Riu Sec, Av. de Vicent Sos Baynat, s/n, 12071 Castelló de la Plana, Spain

## Introduction

Intensive agriculture has put great pressure on populations of beneficial arthropods such as natural enemies and pollinators, especially through adverse effects of pesticide use and the impact on resources in the agricultural landscape, i.e., the reduction of suitable habitats for foraging and nesting sites. The main associated consequences include the decline of biological diversity and delivery of the ecosystem services of biological control and pollination; as a subsequent result, the sustainability of agro-ecosystems is undermined [1,2,3,4,5,6,7,8,9].

Sustainable agronomic practices such as management of field margins and mid-field strips with selected flower plants, cover crops, banker plants, uncultivated areas (set aside), headlands, and hedges can create suitable habitats that provide food and shelter to pollinators and to natural enemies of insect pests in disturbed agro-ecosystems [9,10,11,12,13,14,15,16,17,18,19,20,21]. 

The successful establishment of such habitats requires a good understanding of the food-web theory with respect to functional plant–arthropod diversity, regulation of herbivore populations by manipulation of bottom-up and top-down effects, and crop pollination. Multiple criteria should be considered regarding the selection of plant species for the semi-natural habitats, such as their soil/climatic requirements, growth habits, flowering periods, nectar and pollen production, and flower structure; their potential to become weeds and threaten crop productivity and native flora biodiversity; and finally the tri-trophic interactions between the plants, pests, and target beneficials. 

The spread and possible dominance of a single or only a few plant species would have a direct impact on the desired insect communities in respect to functional biodiversity. Indeed, a narrow plant species selection could support conservation of a certain pollinator group or species in the target area [12,13,22]. In the study by Carvell et al. (2007) [22] a legume-based pollen and nectar flower mix targeted to enhance bumble bee populations in the U.K. could quickly provide a highly attractive forage resource for bumble bees, including rare long-tongued species, whereas a diverse mixture of native wild flowers could attract more of the shorter-tongued *Bombus* spp. and provide greater continuity of forage resources, especially early in the season. In the case of natural enemies, it is widely recognized that increasing biodiversity per se is no guarantee of pest suppression [23]. In general, the key to effective biological control may be tactics that enhance the relative abundance of the most effective natural enemies within the community of natural enemies [24]. Communities are usually characterized as having one or a few species which are relatively abundant (numerically dominant), while the majority of the members of the assemblage are relatively scarce. Moreno et al. (2010) [25] suggested that the success of management strategies by conservation biological control may be dependent on the identification of both highly abundant and scarce natural enemies to determine which assemblage is likely to work best. Nevertheless, the established plant species could possibly attract herbivorous pest species for the crop, higher-order predators/hyperparasitoids, or plant diseases [26].

Specificity on flower species as well as interactions between pollinator species at food searching, especially between managed honey bees and wild bees, are also aspects to be looked at in their habitat creation. Specificity on one or a few particular plant species is an attribute recognized in many insect pollinators when visiting a given foraging bout because floral consistency reduces handling time [27,28,29,30]. However, the fidelity between the pollinator species and plant species from one year to the next can be affected by many parameters such as the degree of specialization of the pollinator species (oligolectic or polylectic, some species being apparently specialist in one year but generalist in another even though the potential plant species pool remains the same) [31,32,33], and the patch area and flower density of the available flowering plant assemblages [34,35]. On the other hand, Shavit et al. (2009) [36] provided evidence that solitary bees did not shift to forage on other flowering plants and did not change their temporal activity pattern as a response to increased foraging by honey bees. 

The benefit of these agronomic practices on the attraction of natural enemies and pollinating insects has been recognized within the flower strip or cover crop in several annual (e.g., cucumber, tomato, watermelon) [37,38,39] or perennial crops (e.g., apple, citrus, olive) [40,41,42]. In fact, the beneficial effect of diversifying fields with nectar-producing floral vegetation on parasitism rates and biological control of insect pests has been supported for several groundcover species and crops by many researchers [14,20,21,43,44]. However, in the case of pollinators, the effect of flower margins on pollination services in terms of yield and fruit quality at farm scale is not yet well established [21]. The value of wild bees in crop pollination was satisfactory in several agro-ecosystems with high populations of wild bees [45,46,47,48], while pollination was insufficient to achieve an acceptable crop yield without managed honey bees in other agro-ecosystems where the abundance or diversity of wild bees was low [45,46,49]. Greater richness of wild bees in agro-ecosystems increases the possibilities for the coexistence of the most effective pollinator species which act complementary to each other during the day or season, alongside extreme climatic conditions and disturbance levels, and would provide sustainability in the agro-ecosystems [30,50,51,52].

This Special Issue aims to focus on good agronomic practices/mitigation measures to sustain and enhance pollinators and natural enemies in terms of plant–arthropod interactions, functional biodiversity, and ecosystem services in cultivated areas. 
